# Variant-specific and cross-reactive antibody responses following COVID-19 booster vaccinations and SARS-CoV-2 breakthrough infections

**DOI:** 10.1038/s41598-026-62635-8

**Published:** 2026-07-15

**Authors:** Iris Medits-Weiss, Dominik Moll, David N. Springer, Stephan W. Aberle, Judith H. Aberle, Karin Stiasny

**Affiliations:** Center for Virology, Medical University of Vienna, Vienna, Austria

**Keywords:** SARS-CoV-2, Omicron BA.5, Booster vaccinations, IgG antibodies, Neutralization, ELISA, Pseudovirus neutralization assay, Variants of concern (VoC), Imprinting, mRNA vaccines, Diseases, Immunology, Microbiology

## Abstract

SARS-CoV-2 has evolved into several genetic variants, all bearing mutations that reduce antibody binding and affect vaccine and treatment effectiveness. Updated COVID-19 vaccines, including bivalent formulations (wild type [WT]/BA.1 or WT/BA.5) and more recent monovalent versions targeting emerging variants such as XBB.1.5, JN.1, KP.2 or LP.8.1, were developed to broaden protection. However, immune imprinting may limit the induction of neutralizing antibodies against strains that differ significantly, even after receiving several variant-specific boosters. A deeper understanding of how booster vaccination reshapes antibody specificity remains essential for rational vaccine design. We examined the antibody response to a bivalent WT/BA.5 booster, focusing on antibody levels and neutralization. Serum samples collected before and after a fourth dose of monovalent WT or bivalent (WT/BA.5) mRNA vaccines were compared with sera from individuals after primary WT infections and Omicron BA.1, BA.2, or BA.5 breakthrough infections. We found that both monovalent and bivalent boosters significantly increased IgG and neutralizing antibodies, but breakthrough infections induced broader cross-reactive responses. Depletion experiments revealed that booster-induced immunity was predominantly mediated by cross-reactive antibodies, with the highest levels after breakthrough infections and the lowest after a primary WT infection. These findings provide functional insights into the antibody specificities associated with imprinting effects following variant-adapted booster vaccination.

**Supplementary Information:**

The online version contains supplementary material available at https://doi.org/10.1038/s41598-026-62635-8.

## Introduction

Since its first appearance in 2019, SARS-CoV-2 has undergone significant genetic evolution, resulting in the development of variants of concern (VOCs) such as Alpha, Delta, and Omicron, as well as several sub-lineages. Mutations in the spike protein affect antibody binding, leading to immune escape, which has significant consequences for vaccine effectiveness and the use of therapeutic monoclonal antibodies^1,2^. The rise of new immune-evasive variants prompted vaccine adaptations that specifically target these variants. Vaccine formulations have been updated using bivalent approaches (e.g., wild type [WT]/BA.1 or WT/BA.5) and newer monovalent vaccines targeting lineages like XBB.1.5, JN.1, KP.2, or LP.8.1. Although these adaptations aimed to enhance neutralization breadth, the phenomenon of immune imprinting may influence the quality and specificity of booster-induced antibody responses. This phenomenon describes the preferential recall and expansion of pre-existing memory B cell responses upon re-exposure to related antigens, potentially limiting the induction of novel strain-specific antibodies^3–7^. Accumulating evidence suggests that immune imprinting occurs across different SARS-CoV-2 infection histories and COVID-19 vaccine platforms, including mRNA, vector- and recombinant protein-based vaccines, supporting the role of antigenic history in shaping subsequent antibody responses^8–11^. Previous studies have demonstrated that bivalent mRNA boosters increase neutralizing antibody titers against circulating Omicron variants, often providing only modest additional breadth compared to repeated ancestral vaccination^12–15^. Consistent with this, several reports indicate that variant-adapted boosters predominantly recall pre-existing cross-reactive memory B cell responses rather than inducing large pools of de novo variant-specific antibodies^16,17^. Moreover, serum depletion studies have shown that repeated exposure to antigenically distinct SARS-CoV-2 variants increasingly promotes cross-reactive and cross-neutralizing antibody responses, including the recognition of antigenically distant coronaviruses that also belong to the subgenus *Sarbecovirus*, which underscores the complex interplay between immune imprinting and antibody breadth^18–20^. Breakthrough infections, particularly with antigenically distinct Omicron subvariants, have been associated with broader neutralization profiles^15,21–23^. These observations underscore the complexity of immune imprinting and highlight the need for further analyses in dissecting antibody specificity within polyclonal serum responses. In the context of repeated COVID-19 vaccination and/or SARS-CoV-2 infection, this may result in amplification of cross-reactive antibodies directed against conserved epitopes rather than robust generation of variant-focused responses. A functional understanding of how booster vaccination reshapes antibody specificity is therefore important for guiding future vaccine strategies.

We evaluated the antibody responses in individuals after a fourth COVID-19 vaccination using either the WT mRNA vaccine or the bivalent mRNA vaccine targeting both WT and BA.5. For comparison, sera from individuals with primary wildtype infections as well as breakthrough infections caused by Omicron subvariants BA.1, BA.2, or BA.5 were included.

Our study specifically investigated the impact of cross-reactive immunity on the total antibody levels and the neutralization capacity against the ancestral D614G and the BA.5 strains. We further dissected the specificity and functionality of the antibody response using receptor-binding domain (RBD) depletion experiments. The central objective was to determine whether booster vaccination or breakthrough infection primarily results in cross-reactive antibodies and/or induces detectable levels of strain-specific RBD-directed antibodies at the serum level.

Our findings reveal that booster vaccinations with either the monovalent or bivalent vaccine increased IgG titers and neutralizing antibodies. Depletion analyses indicated that the majority of these responses were predominantly mediated by cross-reactive RBD-specific antibodies. confirming their central role in virus neutralization. Breakthrough infections were associated with a higher proportion of cross-reactive antibodies, whereas primary WT infection showed the lowest contribution to variant-reactive responses. Overall, vaccination and breakthrough infection mainly resulted in the production of cross-reactive antibodies, rather than producing substantial new variant-specific responses.

## Methods

### Serum samples

Human serum samples were sent to the Center for Virology at the Medical University of Vienna for diagnostic purposes, and were obtained from anonymized collections that had previously been described in published studies^24–28^. Relevant demographic and vaccination data were available from these studies and are summarized in Supplementary Tables 1–3. All vaccinated participants had received mRNA-based COVID-19 vaccines. All methods were carried out in accordance with relevant guidelines and regulations and the Declaration of Helsinki. The use of anonymized residual diagnostic material was approved by the Ethics Committee of the Medical University of Vienna, Austria (EK1291/2021). The requirement for written informed consent was waived in accordance with national legislation and institutional requirements. For functional depletion and neutralization assays, serum samples were combined into defined pools consisting of equal-volume contributions from all individual donors within each cohort (Table 1). The number of individual sera (i_sera) per pool ranged from 7 to 27 individuals. Pooling was performed to ensure sufficient serum volume for repeated depletion and neutralization experiments and to obtain robust cohort-level functional readouts. Pre-booster samples were collected immediately prior to administration of the fourth vaccine dose, whereas post-booster samples were collected after vaccination as indicated in Table 1 and Supplementary Tables 1–3.

### Recombinant proteins/ antigens

Recombinant proteins were produced in Drosophila S2 cells using the pT389 vector (kindly provided by Felix Rey, Institut Pasteur) in the Drosophila Expression System (Invitrogen), as described previously^29^. The vector encodes the spike protein or RBD of SARS-CoV-2 (ancestral D614G or BA.5 variant; GeneArt/Thermo Fisher), an enterokinase cleavage site, and a C-terminal double strep tag. Stably transfected cells were selected with blasticidin, and expression was induced with CuSO₄. The supernatants were collected after 7–10 days. Recombinant proteins were purified via Streptactin-affinity chromatography (IBA GmbH, Göttingen, Germany) following the manufacturer’s protocol.

### ELISA

96-well microtiter plates (Nunc) were coated overnight at 4 °C with 25 ng/well of strep-tagged spike protein or RBD in carbonate buffer. After blocking with 1% BSA in PBS pH 7.4, 0.05% Tween, serial dilutions of serum samples in ELISA buffer (PBS, 2% lamb serum, 2% Tween 20) were added and incubated for 1 h at 37 °C. Human IgG was detected using HRP-conjugated goat anti-human IgG (Thermo Fisher). Titers were calculated via four-parameter logistic regression in GraphPad Prism 10 (GraphPad Software Inc.), using a positive control on each plate. The cut-off was defined as the mean absorbance of 18 pre-pandemic sera at the starting dilution (1:100) plus three standard deviations and was used to determine endpoint titers. Samples were tested in duplicates; mean titers were used for analysis. Statistical comparisons were performed by ANOVA using log-transformed titers, with *p* < 0.05 considered significant.

### Depletions

200 µg recombinant RBD were loaded onto pre-equilibrated Strep-Tactin XT spin columns (IBA GmbH, Göttingen, Germany) using binding buffer (50 mM NaH₂PO₄, 300 mM NaCl, 0.05% Tween 20, pH 8.0). Columns were centrifuged at 700 g for 30 s, and the flow-through was reapplied six times. After washing, columns were equilibrated with depletion buffer (PBS, 0.1% BSA, pH 7.4). Serum samples (1:10) were added and incubated for 10 min at room temperature. Depletion efficiency was verified by IgG ELISA using the corresponding antigen. Mock-depleted control samples were included to account for nonspecific effects associated with the depletion procedure. Mock depletion was carried out using PBS under the same experimental conditions as the antigen-specific depletions. Additional depletion data are provided in Supplementary Tables 4 and Supplementary Fig. 1.

### Pseudovirus neutralization assay

A murine leukemia virus (MLV)-based pseudovirus neutralization assay was used to assess the neutralizing antibody responses against SARS-CoV-2 spike variants, as described previously^30^. Pseudoviruses were produced by co-transfecting HEK293T cells (ATCC CRL-11268) with plasmids for MLV Gag-Pol, a luciferase reporter gene plasmid (kindly provided by Dr. Jean Kaoru Millet^31^, and a plasmid coding for the SARS-CoV-2 spike protein. After 48 h, supernatants were harvested and filtered (0.45 μm) and stored at − 80 °C. Heat-inactivated sera were serially diluted, incubated with pseudovirus particles for 1 h at 37 °C before adding to HEK293T/hACE2 + TMPRSS2 cells (BioCat). After 72 h, luciferase activity was measured using a Luciferase Assay System (Promega), and luminescence was measured in relative light units (RLU) using a microplate reader (Synergy/HTX from BioTek). Neutralization titers were calculated as the serum dilution causing a 50% reduction in relative luminescence units (RLU) compared to pseudovirus-only controls, using non-linear regression analysis in GraphPad Prism. The lower limit of detection of the assay corresponded to the starting serum dilution (1:30).

### Statistical analysis

Statistical analyses were performed with GraphPad Prism 10.2.3 (GraphPad Software Inc.).

For validation of the pooling approach, mean titers of individual sera were compared with pooled serum titers using unpaired two-tailed t-test. To evaluate the effect of booster vaccination on antibody responses (before and after vaccination), paired two-tailed t-tests were performed. For comparisons of independent groups, log-transformed data were analysed by one-way ANOVA with Dunnett’s post-hoc test, as specified for each analysis. Titers below the detection limit of the assay were assigned a value of 50% of this limit for data analysis. P-values < 0.05 were considered significant.

## Results

### Validation of serum pooling

To assess the effect of prior SARS-CoV-2 exposure on the patterns of antibody responses after a bivalent booster vaccination, we analyzed serum samples collected before and after a fourth vaccination. The booster was either the mRNA vaccine targeting the original virus (WT) or the bivalent mRNA vaccine targeting both the WT and the Omicron BA.5 variant (WT/BA.5). In addition, we included serum samples from individuals with primary WT infections and those with breakthrough (BT) infections caused by the Omicron variants BA.1, BA.2, and BA.5 (BT-BA.1, BT-BA.2, and BT-BA.5).

**Fig. 1:**
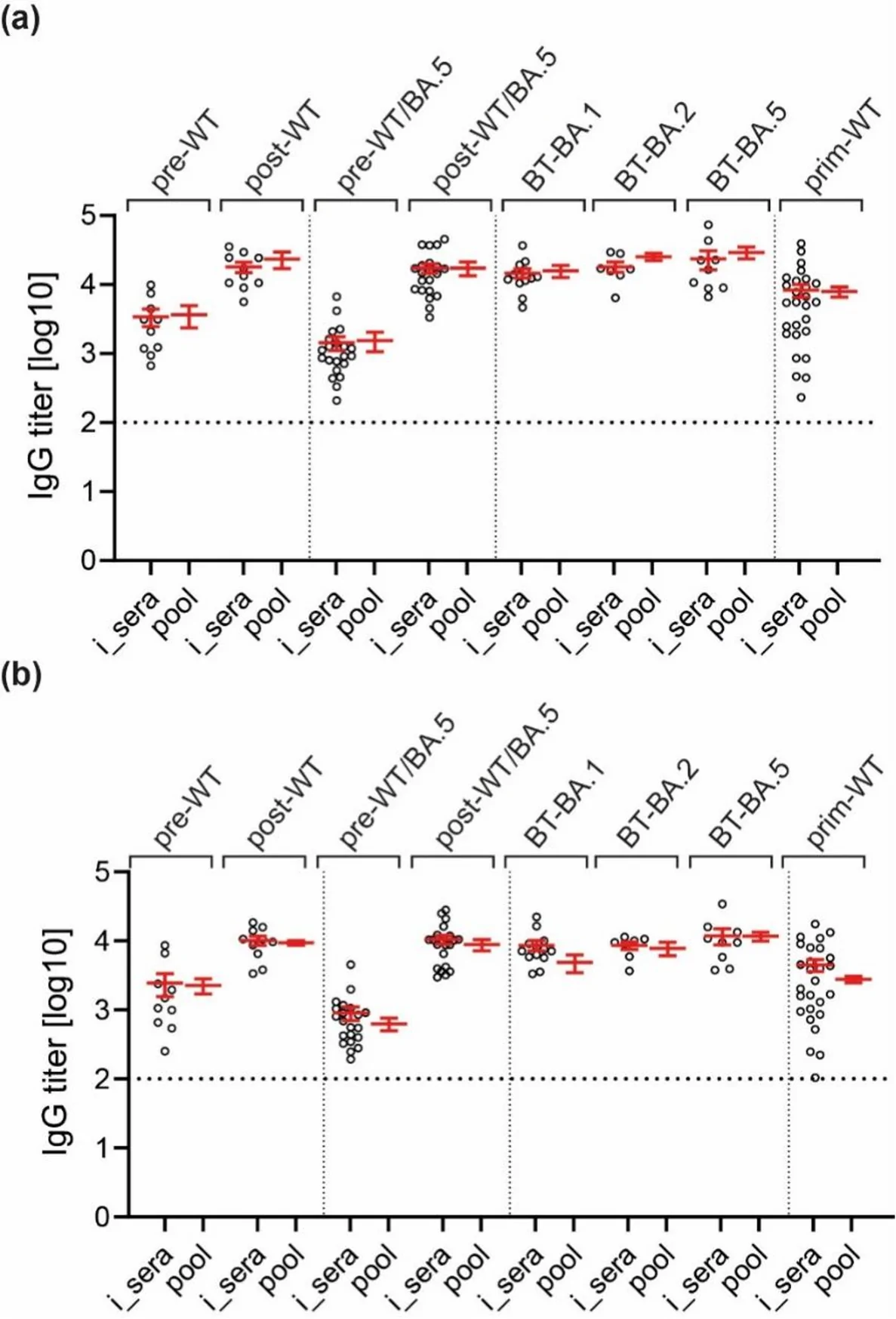
Validation of serum pooling strategy for spike-specific ELISA IgG measurements. Spike-specific IgG ELISA titers obtained from individual sera compared to titers measured in the corresponding serum pools using the D614G spike protein (A) and the BA.5 spike protein (B) as antigens. Each pool represents equal-volume contributions from all individual donors listed in Table 1. Data are presented as mean and standard error of the mean (SEM), with individual serum values indicated by open circles. Results are based on at least two independent replicates. Mean titers of individual sera and their corresponding pooled serum titers showed no significant differences within each group, as determined by unpaired t-test (GraphPad Prism 10.2.3). Abbreviations: BT, breakthrough infection; i_sera, individual sera; prim, primary infection, WT, wild type.

Given the substantial serum volumes required for depletion and neutralization assays, samples from each cohort were combined into defined pools (7–27 individual donors per pool). To verify that pooling did not bias antibody measurements, we quantified virus-specific IgG antibodies directed against the spike protein of D614G and BA.5 by ELISA and directly compared the spike-specific IgG titers from individual sera with those of the corresponding pools. As shown in Fig. 1, the mean ELISA IgG titers obtained with the individual sera closely matched those obtained with the pools using WT and BA.5 spike proteins as antigens, supporting their use for subsequent functional analyses.

**Table 1:** Composition of serum pools and cohort characteristics.

Pool	Immunization status	No. of i_sera	Median age (range)	Sex f/m	dpV^1^/dpI^2^/dpSO^3^ Media (range)
pre-WT	3x WT vaccinated^5^	10	54 (39–65)	6/4	247 (156–268)^1, 4^
post-WT	4x WT vaccinated^5^	10	54 (39–65)	6/4	22 (16–30)^1^
pre-WT/BA.5	3x WT vaccinated^5^	21	50 (27–63)	14/7	302 (231–332)^1, 4^
post-WT/BA.5	4th dose bivalent WT/BA.5^5^	21	50 (27–63)	14/7	21 (21–28)^1^
BT-BA.1	3x WT vaccinated^5^ + BA.1 infection	13	38 (21–77)	8/5	20 (13–36)^2^
BT-BA.2	3x WT vaccinated^5^ + BA.2 infection	7	52 (25–83)	6/1	27 (16–35)^2^
BT-BA.5	3x WT vaccinated^5^ + BA.5 infection	9	56 (32–65)	6/3	24 (22–36)^2^
prim-WT	severe primary WT infection^6^	27	52 (20–90)	6/21	19 (9–25)^3^

i_sera, individual sera; WT, wild type; BT, breakthrough infection; prim, primary infection. ^1^ dpV, days post vaccination. ^2^ dpI, days post infection. ^3^ dpSO, days post symptom onset. ^4^ Pre-booster samples were collected immediately prior to administration of the fourth vaccine dose. ^5^ Vaccinated individuals received mRNA vaccines only. ^6^ All patients in this cohort were hospitalized. Equal volumes of individual sera were combined to generate each serum pool.

### Quantification of spike-specific IgG antibodies and virus neutralization in serum pools

**Fig. 2:**
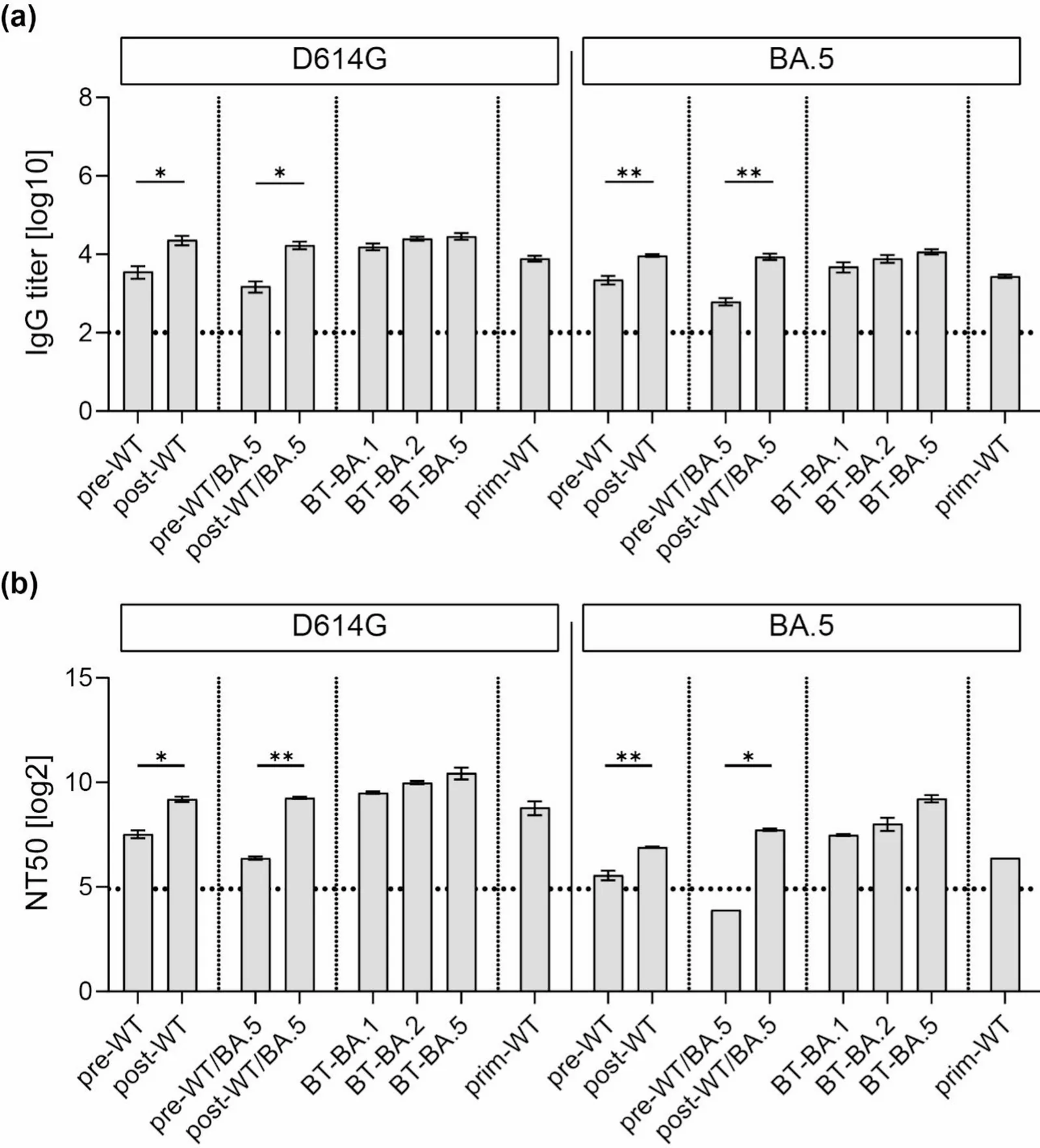
Spike-specific IgG responses and neutralizing activity of serum pools following booster vaccination or breakthrough infection. (a) Spike-specific IgG ELISA titers measured in defined serum pools using D614G (left) and BA.5 (right) spike proteins as antigens (b) Neutralizing antibody titers (NT50) of mock-depleted serum pools against D614G (left) and BA.5 (right) pseudotyped particles. Each pool represents equal-volume contributions from the individual donors listed in Table 1.Error bars represent the standard error of the mean (SEM) of at least two independent experiments. Dashed horizontal lines indicate the assay cut-off. Asterisks indicate significant differences between two groups (measured by paired t-test): *p < 0,05, **p < 0.01. Samples below the detection limit were assigned a value equal to half of the cut-off for statistical analysis.

To analyse the serum antibody response before and after booster vaccination, we quantified total spike-specific IgG and neutralization titers (NT50) against an ancestral D614G strain and a BA.5 strain (Fig. 2). As shown in Fig. 2a, the booster vaccination with either the monovalent or bivalent WT/BA.5 vaccine significantly increased D614G- and BA.5-specific IgG titers to similar levels as observed with breakthrough infections. Overall, BA.5 spike-specific IgG titers were lower than those against the ancestral D614G spike, but without statistical significance (Table 2).

Consistent with the IgG results, the neutralizing activity increased significantly upon monovalent (pre-WT vs. post-WT) and bivalent (pre-WT/BA.5 vs. post-WT/BA.5) vaccination, but neutralization of BA.5 was significantly reduced compared to D614G in all groups (Fig. 2b; Table 2). The titers obtained after booster vaccinations were also lower than those detected after BT infections, for both the D614G and the BA.5 strain. However, significant differences were detected between some of the groups, as summarized in Table 3. Notably, the pool before WT booster vaccination (pre-WT) had higher IgG and NT titers than the pool before bivalent WT/BA.5 booster vaccination (pre-WT/BA.5), with a significant difference in neutralization (*p* = 0.0014); however, after the fourth vaccine dose, antibody levels were similar in both groups.

**Table 2:** Statistical comparison of WT and BA.5 spike-specific IgG titers and neutralizing antibody titers within each serum pool using one-way ANOVA followed by Dunnett’s multiple comparisons test.

pools	Statistical differences between WT and BA5 reactivity	
	IgG Titers (*p* values)	NT50 - GMT (*p* values)
pre-WT	ns (*p* = 0.9971)	**** (*p* < 0.0001)
post-WT	ns (*p* = 0.5903)	**** (*p* < 0.0001)
pre-WT/BA.5	ns (*p* = 0.6116)	**** (*p* < 0.0001)^#^
post-WT/BA.5	ns (*p* = 0.8717)	*** (*p* = 0.0008)
BT-BA.1	ns (*p* = 0.0787)	**** (*p* < 0.0001)
BT-BA.2	ns (*p* = 0.0888)	**** (*p* < 0.0001)
BT-BA.5	ns (*p* = 0.4483)	** (*p* = 0.0084)
prim-WT	ns (*p* = 0.5314)	**** (*p* < 0.0001)

^#^ Values below the detection limit (ELISA < 100, NT < 30) were set to half the cut-off for statistical analysis.

ns, not significant.

**Table 3:** Statistical comparison of neutralizing antibody titers (NT50) between booster-vaccinated and breakthrough infection serum pools determined by one-way ANOVA followed by Dunnett’s multiple comparisons test.

	D614G-GMT (*p* values)			BA.5-GMT (*p* values)		
	BT-BA.1	BT-BA.2	BT-BA.5	BT-BA.1	BT-BA.2	BT-BA.5
post-WT	ns (*p* = 0.9895)	ns (*p* = 0.1805)	** (*p* = 0.0081)	ns (*p* = 0.5594)	* (*p* = 0.0213)	**** (*p* < 0,0001)
post-WT/BA.5	ns (*p* = 0.9993)	ns (*p* = 0.2844)	* (*p* = 0.0139)	ns (*p* = 0.9988)	ns (*p* = 0.9983)	** (*p* = 0.0013)

ns, not significant.

### Homologous and heterologous RBD-depletion specificity

**Fig. 3:**
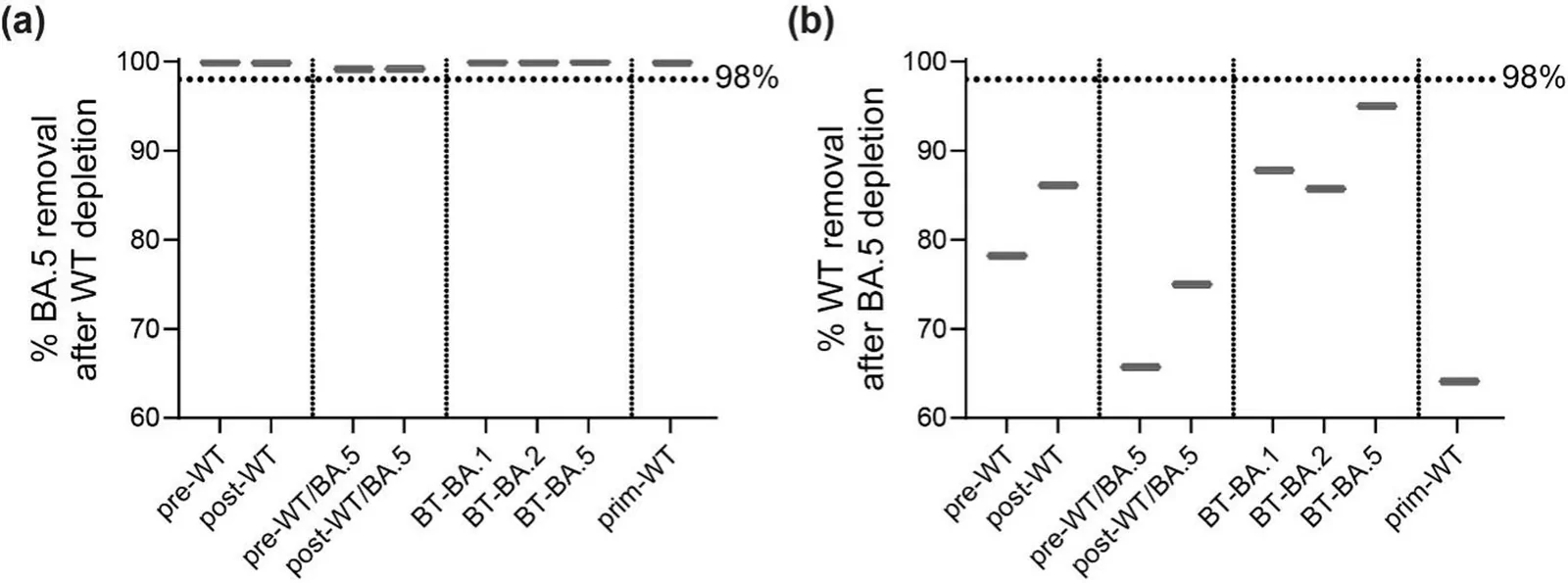
Percentage removal of WT- and BA.5-RBD–specific antibodies following heterologous RBD depletion. (a) Percentage removal of BA.5-RBD reactivity after depletion with WT-RBD. (b) Percentage removal of WT-RBD reactivity after depletion with BA.5-RBD. Each pool consists of equal-volume contributions from the individual donors listed in Table 1. Each data point represents the percent reduction in ELISA IgG binding compared to mock-depleted control pools. The dotted horizontal line indicates near-complete depletion (≥ 98%). Vertical dashed lines separate vaccination-only groups from breakthrough infection groups. WT, wild type; BT, breakthrough infection.

As neutralizing antibodies mainly target the RBD^32^, we tested whether the booster vaccination with the bivalent WT/BA.5 vaccine can induce new strain-specific antibodies or results in an enhancement of cross-reactive antibodies. To this end, we depleted the serum pools (Fig. 2) with the WT- and BA.5-RBD.Representative depletion ELISA curves and a summary of undepleted, mock-depleted, and antigen-depleted ELISA and neutralization titers are provided in Supplementary Tables 4 and Supplementary Fig. 1. IgG ELISAs with RBDs as antigens revealed the complete removal of antibodies recognizing the respective homologous RBD (> 98% for all pools), as shown in Supplementary Fig. 2. Depletion with the WT-RBD also resulted in a near-complete depletion of BA.5-RBD reactivity across all groups, indicating that the majority of BA.5-RBD–binding antibodies were cross-reactive and recognized epitopes shared with WT-RBD (Fig. 3a). In contrast, after serum depletion with BA.5-RBD, we found residual reactivity with the WT-RBD, indicating the presence of WT-RBD-specific antibodies not recognized by the BA.5-RBD (Fig. 3b), with a higher degree of depletion of pools from BA.5 breakthrough infections (BT-BA.5: 95%) than after the bivalent booster (post-WT/BA.5: 75%), demonstrating asymmetric cross-reactivity between WT-RBD and BA.5-RBD-directed antibodies. The percentage depletion across all serum pools is summarized in Supplementary Table 5.

### Impact of RBD depletion on spike-specific IgG antibody titers

**Fig. 4:**
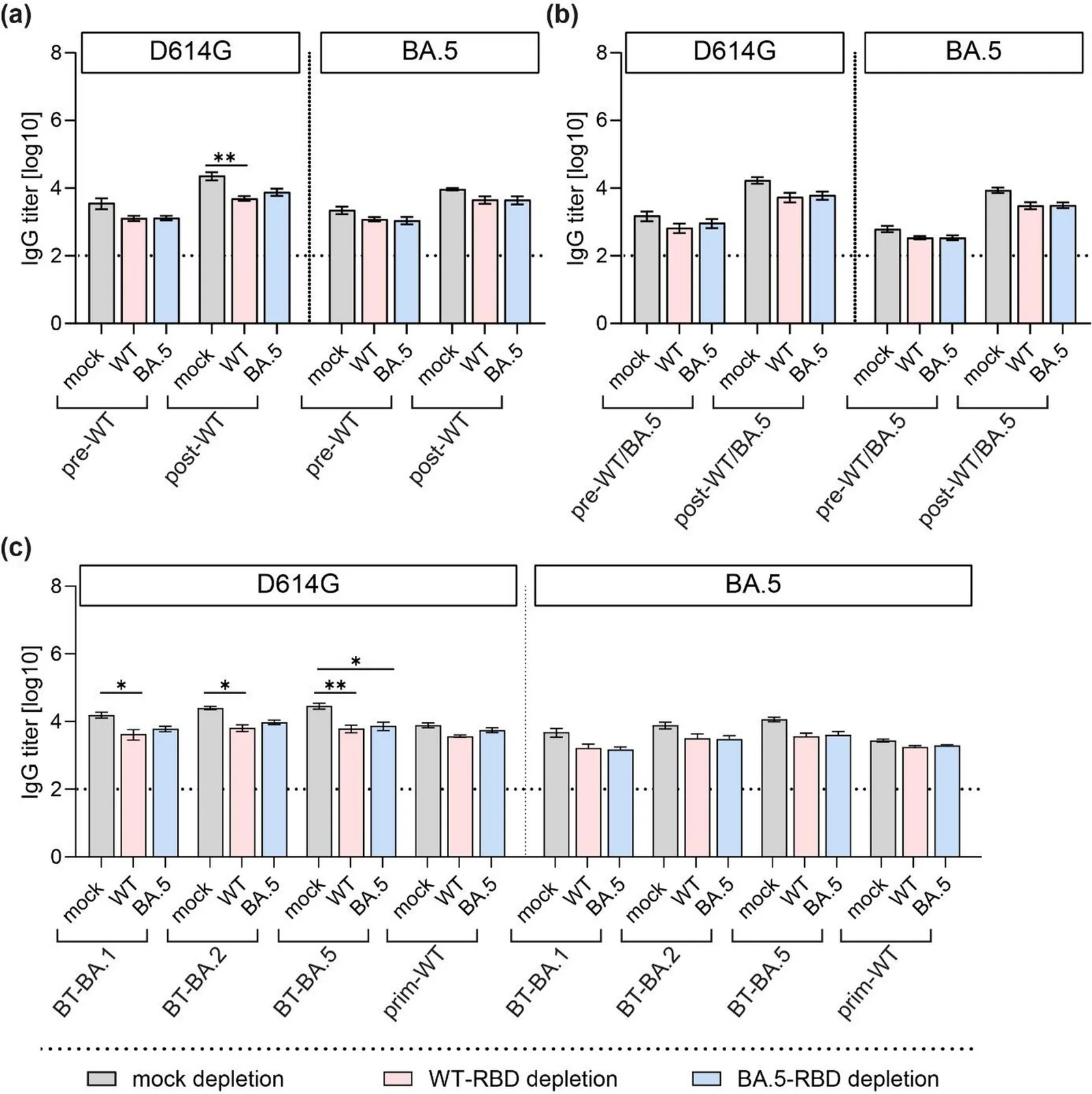
Contribution of WT- and BA.5-directed RBD antibodies to spike-specific IgG reactivity following depletion. Serum pools were depleted with WT-RBD (red) or BA.5-RBD (blue) and subsequently analyzed for residual spike-specific IgG binding to full-length D614G spike (left) and BA.5 spike (right) proteins by ELISA. Each pool represents equal-volume contributions from the individual donors listed in Table 1. Error bars represent the standard error of the mean (SEM) of at least two independent experiments. Dashed horizontal lines indicate the assay cut-off. Asterisks indicate significant differences to mock-depleted pools as determined by one-way ANOVA followed by Dunnett’s multiple comparisons test on log-transformed titers using GraphPad Prism 10.2.3 (*p < 0.05, **p < 0.01).

To assess the contribution of RBD-specific antibodies to the overall spike-specific IgG response, we performed ELISAs with a WT and BA.5 spike protein (Fig. 4). Depletion with either WT-RBD or BA.5-RBD proteins led to reductions in IgG titers against D614G and BA.5 spike proteins across all groups, consistent with the dominant role of RBD-directed antibodies within the spike-specific response. Depletion with either WT-RBD or BA.5-RBD resulted in reduced IgG titers against both D614G and BA.5 spike proteins across all groups. However, a statistically significant decrease in D614G spike reactivity was observed after WT-RBD depletion in sera collected following monovalent WT booster vaccination (post-WT; Fig. 4a) and in individuals with breakthrough infections caused by Omicron subvariants BA.1, BA.2, and BA.5 (BT-BA.1, BT-BA.2, and BT-BA.5). Depletion with BA.5-RBD led to a significantly reduced D614G spike-specific IgG only in sera from individuals with BA.5 breakthrough infection (BT-BA.5; Fig. 4c). These findings indicate that, while RBD-directed antibodies broadly contribute to spike reactivity across groups, the relative contribution of BA.5-RBD–reactive antibodies to D614G spike recognition are most pronounced following BA.5 breakthrough infection. The magnitude of reduction was modest, suggesting that additional non-RBD-directed antibodies may contribute to overall spike reactivity.

### Impact of RBD depletion on neutralization

**Fig. 5:**
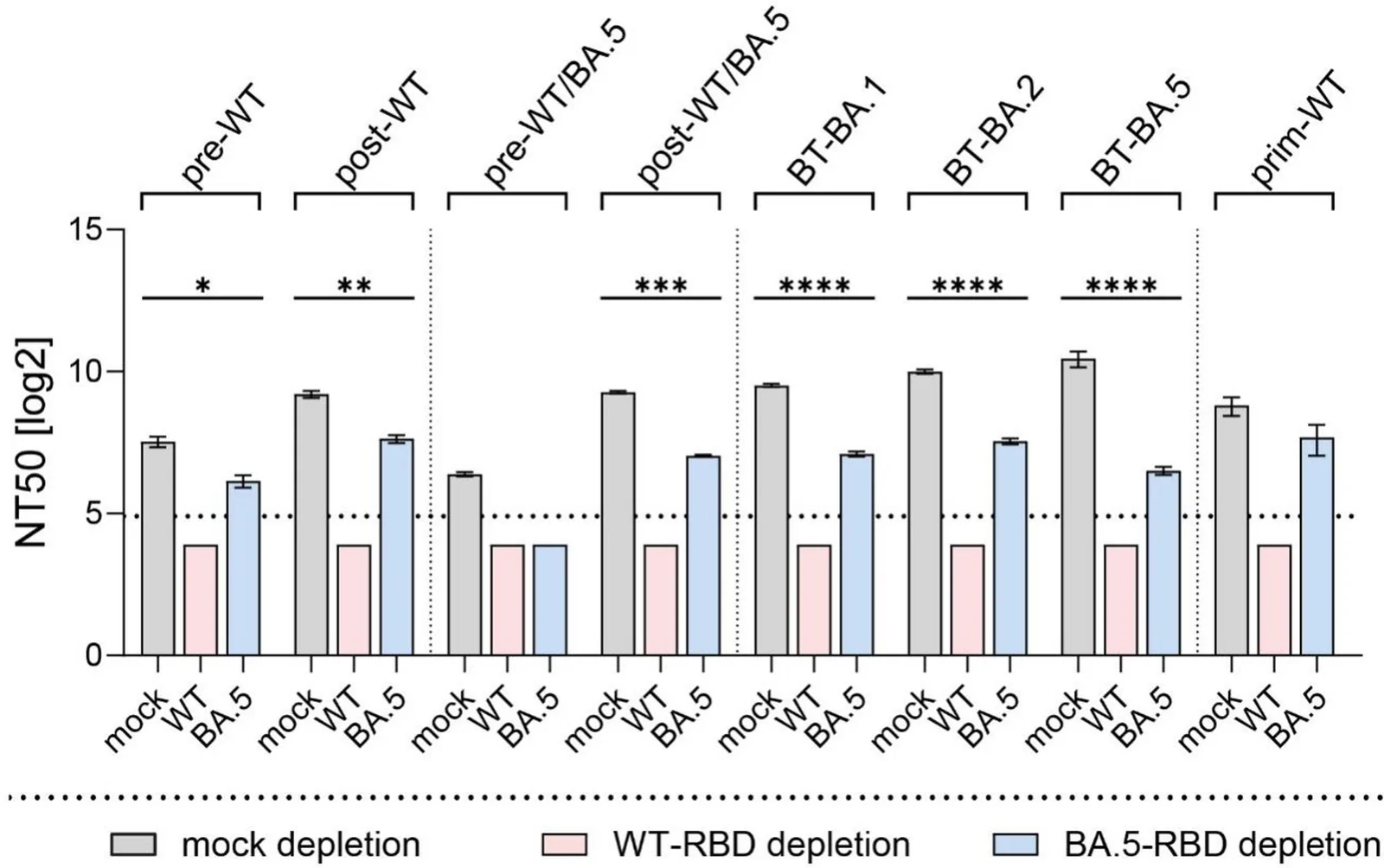
Impact of RBD-specific antibody depletion on D614G neutralization capacity. Serum pools were depleted with WT-RBD (red) or BA.5-RBD (blue) and subsequently tested for residual neutralizing activity (NT50) against D614G spike–pseudotyped particles. Each pool represents equal-volume contributions from the individual donors listed in Table 1. Error bars represent the standard error of the mean (SEM) of at least two independent experiments. Dashed horizontal line indicates the assay cut-off. Statistical comparisons were performed relative to mock-depleted control pools using one-way ANOVA followed by Dunnett’s multiple comparisons test on log-transformed NT50 titers (*p < 0.05, **p < 0.01) using GraphPad Prism 10.2.3.

We finally determined the impact of the different RBD-specific antibodies on serum neutralization. Depletion with the homologous RBD protein (WT-RBD for D614G neutralization, BA.5-RBD for BA.5 neutralization) resulted in a marked reduction of neutralizing activity below the detection limit, confirming the central contribution of RBD-directed antibodies to variant-specific neutralization. We also observed a similar effect for BA.5 neutralization after depletion with the WT-RBD, consistent with the predominance of cross-reactive RBD-specific antibodies. As these results were consistently negative in all groups and did not reveal group-specific differences, the data are provided in Supplementary Fig. 3. In contrast, heterologous depletion with BA.5-RBD led to a partial reduction of D614G neutralization, with significant differences compared to the non-depleted control (Fig. 5). This reduction was more pronounced in sera obtained after breakthrough infections (BT-BA.1, BT-BA.2, BT-BA.5) or bivalent boosters (post-WT/BA.5). Monovalent sera before (pre-WT) and after booster vaccination (post-WT) showed smaller effects. Neutralization by sera from individuals obtained after primary WT infection (prim-WT) were largely unaffected, indicating limited induction of BA.5-directed antibody responses without additional exposures to other strains/variants.

## Discussion

The evolution of SARS-CoV-2, particularly the emergence of Omicron subvariants, has posed a challenge to the effectiveness of vaccines and the durability of immunity. While the original WT vaccines provided strong protection against early variants, Omicron and its sublineages were able to evade pre-existing neutralizing antibodies. Updated bivalent mRNA vaccines, which include both ancestral and Omicron components, aimed to broaden protection, but their ability to generate antibodies that specifically target new variants appears to be rather limited due to immunological imprinting, whereby previous exposure to ancestral antigens shapes and biases subsequent immune responses^7,33,34^.

Our analysis demonstrates that both monovalent WT and bivalent mRNA booster vaccinations markedly increased spike-specific IgG titers and neutralizing activity against D614G and BA.5 (Fig. 2), confirming their robust boosting potential. Nevertheless, antibody levels against BA.5 were consistently lower than those against D614G. The same pattern was observed for the neutralisation of BA.5, which was reduced compared to D614G (Table 2). It should be noted that the pre-booster samples were collected immediately prior to administration of the fourth vaccine dose. Consequently, differences between pre- and post-booster samples likely reflect both the effects of booster vaccination and temporal changes associated with antibody waning and affinity maturation^35,36^. Our findings indicate that the bivalent vaccine, despite containing an Omicron BA.5 component, did not elicit stronger antibody responses against BA.5 than the monovalent WT booster. Our results align with those of other studies which have reported no or little advantage of bivalent mRNA vaccines over monovalent WT boosters in inducing variant-specific neutralizing antibody responses^37–44^.

Our study also provides an important comparison between mRNA vaccine-induced immunity and hybrid immunity resulting from a combination of mRNA vaccination and natural infection. Antibody levels after booster vaccination were generally lower than those observed following breakthrough infections, which is consistent with recent reports showing that hybrid immunity is often associated with broader and more robust immune responses than vaccination alone^39,41,42^. However, even after breakthrough infection, neutralization titers against more recent Omicron subvariants remained low. Differences in antibody subclass distributions may also contribute to the distinct patterns observed after vaccination and breakthrough infection. Recent studies have reported increased IgG4 responses following repeated mRNA vaccination, whereas natural infection and other vaccine platforms may induce different subclass profiles^45^.

The RBD of the SARS-CoV-2 spike is the main target of neutralizing antibodies, making its antigenic properties central to immunity. Depletion experiments revealed that RBD-binding antibodies accounted for most spike-specific and neutralizing activity, confirming the RBD as the dominant antigenic site^46–48^. In our study, WT-RBD depletion completely removed BA.5-RBD reactivity, demonstrating substantial cross-reactivity between ancestral and Omicron BA.5 RBDs. In contrast, residual WT-RBD reactivity after BA.5-RBD depletion revealed asymmetric cross-recognition, with not all ancestral antibodies binding BA.5. Notably, depletion patterns differed between vaccination and breakthrough infection. BA.5 breakthrough infections led to a higher degree of cross-reactive antibody responses than bivalent vaccinations, indicating that infection may promote broader antibody repertoires. These findings support the concept that antigenic exposure through infection can expand cross-reactive memory responses more effectively than vaccination alone, consistent with recent studies demonstrating imprinting-driven recall of cross-reactive antibody responses^16,17^. Although statistically significant, the relatively small decrease in antibody reactivity suggests that epitopes outside the RBD also contribute to total spike reactivity.

Depletion of RBD-specific antibodies significantly reduced IgG binding and neutralizing activity across all groups, confirming the central role of the RBD in mediating neutralization. Homologous depletion resulted in the strongest reduction in neutralizing activity, while heterologous depletion revealed partial cross-neutralization. While low levels of BA.5-specific antibodies cannot be excluded, the depletion data indicate that the majority of detectable BA.5-neutralizing activity is mediated by antibodies recognizing epitopes shared between WT and BA.5. The incomplete reduction of D614G neutralization following BA.5-RBD depletion further indicates that antibodies targeting ancestral epitopes remain dominant. Similar observations have been reported in previous studies investigating cross-neutralizing antibody responses^49,50^. Similarly, several serum depletion studies have demonstrated that repetitive exposure to antigenically distinct SARS-CoV-2 variants promotes increasingly cross-reactive and cross-neutralizing antibody responses^18–20^. In particular, Liang et al. reported that boosting with updated SARS-CoV-2 antigens broadens cross-neutralizing activity, extending even to antigenically distant sarbecoviruses^19^. Although our study focused on WT and BA.5 responses, our findings are consistent with these observations and further support the predominance of cross-reactive antibody responses following repeated antigenic exposure. However, given the use of pooled sera and serum-level depletion, the data do not allow discrimination between newly induced strain-specific antibodies and expansion of pre-existing cross-reactive memory responses at the clonal level. Our study was not designed to directly quantify de novo B-cell induction but to assess functional serum-level antibody reactivity. Although the use of pooled sera did not allow analyzing inter-individual variability, it enabled a robust functional characterization of cohort-level antibody responses by depletion and neutralization assays that require substantial serum volumes. Neutralizing antibodies can also target epitopes within the N-terminal domain (NTD) of the spike protein^51,52^. Therefore, we cannot exclude that NTD-directed antibodies may have additionally contributed to the observed neutralizing activity. Furthermore, authentic virus neutralization assays were not performed. The pseudovirus assay used in this study has recently been validated against live-virus neutralization assays, demonstrating a close correlation between the two approaches^30^.

The aim of variant-adapted monovalent vaccines, which present the spike protein of the currently circulating variant, is to broaden and redirect the antibody response. However, immune imprinting remains a key factor in shaping the humoral response^4–7,53^. In line with the literature, we demonstrate that immune imprinting influences both the magnitude and breadth of the immune response. This was also evident in studies, which characterized the B-cell repertoire after vaccinations and/or infections^9,11,22–55^.

Taken together, the analysis of well-characterized sera from bivalent vaccinees with detailed immunization histories provides important insights into the impact of immune imprinting on antibody responses. Ongoing research is investigating whether repeated Omicron exposures can overcome ancestral imprinting. However, results remain inconclusive so far^23,56^. Although our study cohorts and the vaccine formulations no longer reflect the current landscape of circulating variants and population immunity, the underlying immunological principles remain highly relevant. In particular, the observed patterns of asymmetric cross-reactivity and immune imprinting are likely to extend to current circulating variants, where the antigenic distance from the ancestral strain is even more pronounced. Consequently, understanding how repeated antigenic exposure shapes cross-reactive antibody responses and immune imprinting remains important for the continued development of COVID-19 vaccines and vaccination strategies.

## Supplementary Information

Below is the link to the electronic supplementary material.

Supplementary Material 1

## Data Availability

The datasets generated and/or analyzed during the current study are available from the corresponding author upon request.
