# Supplementary material for: Variant-specific and cross-reactive antibody responses following COVID-19 booster vaccinations and SARS-CoV-2 breakthrough infections
Source: Sci Rep. 2026 Jul 15;16:33145. doi: 10.1038/s41598-026-62635-8 (PMC13683043; doi:10.1038/s41598-026-62635-8)
Supplement: Supplementary file 1 [file 41598_2026_62635_MOESM1_ESM.docx]

**Supplementary Table 1: Characteristics of serum samples included in the vaccinated cohorts.**
Individual serum samples contributing to the pre-WT, post-WT, pre-WT/BA.5, and post-WT/BA.5 pools, including vaccination status and sampling intervals. All individuals received mRNA-based COVID-19 vaccines. Equal volumes of individual sera were used for pool generation

| **Serum** | **sex** | **age** | | **Vacc 4** | **dpVacc3** | **pool** | **dpVacc4** | **pool** |
| --- | --- | --- | --- | --- | --- | --- | --- | --- |
| Vac_01 | f | 53 | | WT, monovalent | 252 | pre-WT | 20 | post-WT |
| Vac_02 | m | 42 | | WT, monovalent | 244 | pre-WT | 16 | post-WT |
| Vac_03 | m | 46 | | WT, monovalent | 208 | pre-WT | 21 | post-WT |
| Vac_04 | f | 65 | | WT, monovalent | 156 | pre-WT | 22 | post-WT |
| Vac_05 | m | 56 | | WT, monovalent | 253 | pre-WT | 24 | post-WT |
| Vac_06 | f | 41 | | WT, monovalent | 220 | pre-WT | 21 | post-WT |
| Vac_07 | m | 39 | | WT, monovalent | 267 | pre-WT | 24 | post-WT |
| Vac_08 | f | 54 | | WT, monovalent | 231 | pre-WT | 30 | post-WT |
| Vac_09 | f | 63 | | WT, monovalent | 268 | pre-WT | 29 | post-WT |
| Vac_10 | f | 58 | | WT, monovalent | 250 | pre-WT | 18 | post-WT |
| Vac_11 | f | 39 | | WT/BA.5, bivalent | 332 | pre-WT/BA.5 | 21 | post-WT/BA.5 |
| Vac_12 | m | 46 | | WT/BA.5, bivalent | 309 | pre-WT/BA.5 | 21 | post-WT/BA.5 |
| Vac_13 | m | 39 | | WT/BA.5, bivalent | 231 | pre-WT/BA.5 | 21 | post-WT/BA.5 |
| Vac_14 | m | 34 | | WT/BA.5, bivalent | 243 | pre-WT/BA.5 | 21 | post-WT/BA.5 |
| Vac_15 | f | 56 | | WT/BA.5, bivalent | 232 | pre-WT/BA.5 | 21 | post-WT/BA.5 |
| Vac_16 | f | 58 | | WT/BA.5, bivalent | 302 | pre-WT/BA.5 | 21 | post-WT/BA.5 |
| Vac_17 | f | 53 | | WT/BA.5, bivalent | 308 | pre-WT/BA.5 | 21 | post-WT/BA.5 |
| Vac_18 | m | 63 | | WT/BA.5, bivalent | 298 | pre-WT/BA.5 | 21 | post-WT/BA.5 |
| Vac_19 | f | 52 | | WT/BA.5, bivalent | 306 | pre-WT/BA.5 | 21 | post-WT/BA.5 |
| Vac_20 | m | 42 | | WT/BA.5, bivalent | 232 | pre-WT/BA.5 | 21 | post-WT/BA.5 |
| Vac_21 | f | 39 | | WT/BA.5, bivalent | 301 | pre-WT/BA.5 | 21 | post-WT/BA.5 |
| Vac_22 | f | 33 | | WT/BA.5, bivalent | 318 | pre-WT/BA.5 | 21 | post-WT/BA.5 |
| Vac_23 | f | 56 | | WT/BA.5, bivalent | 298 | pre-WT/BA.5 | 21 | post-WT/BA.5 |
| Vac_24 | f | 45 | | WT/BA.5, bivalent | 287 | pre-WT/BA.5 | 21 | post-WT/BA.5 |
| Vac_25 | f | 57 | | WT/BA.5, bivalent | 304 | pre-WT/BA.5 | 21 | post-WT/BA.5 |
| Vac_26 | f | 50 | | WT/BA.5, bivalent | 307 | pre-WT/BA.5 | 21 | post-WT/BA.5 |
| Vac_27 | f | 56 | | WT/BA.5, bivalent | 298 | pre-WT/BA.5 | 21 | post-WT/BA.5 |
| Vac_28 | f | 51 | | WT/BA.5, bivalent | 306 | pre-WT/BA.5 | 21 | post-WT/BA.5 |
| Vac_29 | m | 56 | | WT/BA.5, bivalent | 243 | pre-WT/BA.5 | 21 | post-WT/BA.5 |
| Vac_30 | m | 28 | | WT/BA.5, bivalent | 328 | pre-WT/BA.5 | 28 | post-WT/BA.5 |
| Vac_31 | f | 27 | | WT/BA.5, bivalent | 330 | pre-WT/BA.5 | 28 | post-WT/BA.5 |
| **Source publications:** | | | Medits et al. (2022), Ref. 22.  Springer et al. (2024), Ref. 24. | | | | | |

**Supplementary Table 2. Characteristics of serum samples included in the breakthrough infection cohorts.**
Individual serum samples contributing to the BA.1, BA.2, and BA.5 breakthrough (BT) infection pools, including vaccination status and sampling intervals. All individuals received mRNA-based COVID-19 vaccines. Equal volumes of individual sera were used for pool generation.

| Serum | sex | | age | infecting variant | dpI | pool |
| --- | --- | --- | --- | --- | --- | --- |
| BT_01 | f | | 54 | BA.1 | 20 | BT-BA.1 |
| BT_02 | f | | 47 | BA.1 | 19 | BT-BA.1 |
| BT_03 | f | | 33 | BA.1 | 18 | BT-BA.1 |
| BT_04 | f | | 40 | BA.1 | 18 | BT-BA.1 |
| BT_05 | m | | 27 | BA.1 | 20 | BT-BA.1 |
| BT_06 | m | | 23 | BA.1 | 20 | BT-BA.1 |
| BT_07 | f | | 25 | BA.1 | 17 | BT-BA.1 |
| BT_08 | m | | 43 | BA.1 | 13 | BT-BA.1 |
| BT_09 | f | | 77 | BA.1 | 30 | BT-BA.1 |
| BT_10 | f | | 34 | BA.1 | 36 | BT-BA.1 |
| BT_11 | m | | 55 | BA.1 | 24 | BT-BA.1 |
| BT_12 | f | | 38 | BA.1 | 23 | BT-BA.1 |
| BT_13 | m | | 21 | BA.1 | 20 | BT-BA.1 |
| BT_14 | f | | 57 | BA.2 | 27 | BT-BA.2 |
| BT_15 | f | | 28 | BA.2 | 22 | BT-BA.2 |
| BT_16 | f | | 60 | BA.2 | 35 | BT-BA.2 |
| BT_17 | f | | 52 | BA.2 | 27 | BT-BA.2 |
| BT_18 | m | | 28 | BA.2 | 28 | BT-BA.2 |
| BT_19 | f | | 83 | BA.2 | 16 | BT-BA.2 |
| BT_20 | f | | 25 | BA.2 | 33 | BT-BA.2 |
| BT_21 | f | | 55 | BA.5 | 24 | BT-BA.5 |
| BT_22 | f | | 57 | BA.5 | 22 | BT-BA.5 |
| BT_23 | m | | 54 | BA.5 | 28 | BT-BA.5 |
| BT_24 | f | | 32 | BA.5 | 24 | BT-BA.5 |
| BT_25 | f | | 38 | BA.5 | 30 | BT-BA.5 |
| BT_26 | f | | 60 | BA.5 | 22 | BT-BA.5 |
| BT_27 | f | | 65 | BA.5 | 36 | BT-BA.5 |
| BT_28 | m | | 56 | BA.5 | 23 | BT-BA.5 |
| BT_29 | m | | 60 | BA.5 | 24 | BT-BA.5 |
| **Source publications:** | | Medits et al. (2022), Ref. 22.  Springer et al. (2024), Ref. 23.  Springer et al. (2024), Ref. 24. | | | | |

**Supplementary Table 3. Characteristics of serum samples included in the primary WT infection cohort.**
Individual serum samples contributing to the primary WT infection pool, including sampling intervals. All patients in this cohort were hospitalized and therefore represent severe primary SARS-CoV-2 infections. Equal volumes of individual sera were used for pool generation.

| Serum | sex | | age | infecting variant | dpSO | pool |
| --- | --- | --- | --- | --- | --- | --- |
| prim_01 | m | | 23 | WT | 10 | prim-WT |
| prim_02 | m | | 33 | WT | 11 | prim-WT |
| prim_03 | m | | 20 | WT | 17 | prim-WT |
| prim_04 | f | | 51 | WT | 25 | prim-WT |
| prim_05 | f | | 45 | WT | 24 | prim-WT |
| prim_06 | m | | 36 | WT | 12 | prim-WT |
| prim_07 | m | | 38 | WT | 16 | prim-WT |
| prim_08 | m | | 32 | WT | 23 | prim-WT |
| prim_09 | m | | 28 | WT | 20 | prim-WT |
| prim_10 | m | | 33 | WT | 20 | prim-WT |
| prim_11 | m | | 52 | WT | 23 | prim-WT |
| prim_12 | m | | 48 | WT | 15 | prim-WT |
| prim_13 | f | | 49 | WT | 15 | prim-WT |
| prim_14 | f | | 68 | WT | 19 | prim-WT |
| prim_15 | m | | 54 | WT | 19 | prim-WT |
| prim_16 | m | | 78 | WT | 21 | prim-WT |
| prim_17 | m | | 57 | WT | 20 | prim-WT |
| prim_18 | m | | 73 | WT | 17 | prim-WT |
| prim_19 | m | | 63 | WT | 9 | prim-WT |
| prim_20 | f | | 72 | WT | 11 | prim-WT |
| prim_21 | m | | 46 | WT | 13 | prim-WT |
| prim_22 | m | | 90 | WT | 19 | prim-WT |
| prim_23 | m | | 57 | WT | 19 | prim-WT |
| prim_24 | m | | 62 | WT | 21 | prim-WT |
| prim_25 | f | | 73 | WT | 21 | prim-WT |
| prim_26 | m | | 75 | WT | 17 | prim-WT |
| prim_27 | m | | 52 | WT | 21 | prim-WT |
| **Source publications:** | | Koblischke et al. (2020), Ref.21.  Graninger et al. (2022), Ref. 20.  Medits et al. (2022), Ref. 22. | | | | |

**Supplementary Table 4** Summary of undepleted, mock-depleted, WT-RBD-depleted, and BA.5-RBD-depleted ELISA and neutralization titers for all serum pools.

| **Pool** | **Depletion** | **IgG Titer  WT-RBD** | **IgG Titer BA.5-RBD** | **IgG Titer D614G Spike** | **IgG Titer  BA.5 Spike** | **NT50 - GMT D614G** | **NT50 - GMT BA.5** |
| --- | --- | --- | --- | --- | --- | --- | --- |
| pre-WT | undepleted | 9789 | 1672 | nd | nd | nd | nd |
|  | mock | 3871 | 960 | 3663 | 2270 | 184 | 47 |
|  | WT-RBD | < cut-off | < cut-off | 1300 | 1217 | <30 | <30 |
|  | BA.5-RBD | 843 | < cut-off | 1328 | 1130 | 70 | <30 |
| post-WT | undepleted | 45146 | 14208 | nd | nd | nd | nd |
|  | mock | 13528 | 5371 | 23343 | 9357 | 586 | 119 |
|  | WT-RBD | 198 | < cut-off | 5006 | 4591 | <30 | <30 |
|  | BA.5-RBD | 1882 | < cut-off | 7767 | 4501 | 197 | <30 |
| pre- WT/BA.5 | undepleted | 1042 | 629 | nd | nd | nd | nd |
|  | mock | 1330 | 592 | 1549 | 629 | 84 | <30 |
|  | WT-RBD | < cut-off | < cut-off | 677 | 345 | <30 | <30 |
|  | BA.5-RBD | 457 | < cut-off | 950 | 346 | <30 | <30 |
| post-WT/BA.5 | undepleted | 19526 | 9251 | nd | nd | nd | nd |
|  | mock | 7778 | 7397 | 17291 | 8831 | 617 | 214 |
|  | WT-RBD | < cut-off | < cut-off | 5520 | 3088 | <30 | <30 |
|  | BA.5-RBD | 1945 | < cut-off | 6219 | 3153 | 131 | <30 |
| BT-BA.1 | undepleted | 32426 | 7718 | nd | nd | nd | nd |
|  | mock | 22306 | 7897 | 15815 | 4873 | 729 | 180 |
|  | WT-RBD | < cut-off | < cut-off | 4355 | 1660 | <30 | <30 |
|  | BA.5-RBD | 2710 | < cut-off | 6149 | 1499 | 137 | <30 |
| BT-BA.2 | undepleted | 28342 | 7684 | nd | nd | nd | nd |
|  | mock | 24846 | 8361 | 25260 | 7851 | 1016 | 256 |
|  | WT-RBD | < cut-off | < cut-off | 6564 | 3221 | <30 | <30 |
|  | BA.5-RBD | 3555 | < cut-off | 9616 | 3022 | 186 | <30 |
| BT-BA.5 | undepleted | 52568 | 18000 | nd | nd | nd | nd |
|  | mock | 26352 | 17232 | 29191 | 11704 | 1374 | 594 |
|  | WT-RBD | < cut-off | < cut-off | 6268 | 3730 | <30 | <30 |
|  | BA.5-RBD | 1305 | < cut-off | 7541 | 4101 | 90 | <30 |
| prim-WT | undepleted | 5790 | 25 | nd | nd | nd | nd |
|  | mock | 2898 | 53 | 7915 | 2768 | 434 | 84 |
|  | WT-RBD | < cut-off | < cut-off | 3702 | 1780 | 36 | <30 |
|  | BA.5-RBD | 1041 | < cut-off | 5633 | 1995 | 192 | <30 |
| nd, not determined | | | | | | | |
| < cut-off, tested negative (below ELISA detection limit) | | | | | | | |

**Supplementary Figure 1:** Representative ELISA depletion curves demonstrating depletion efficiency and specificity. Absorbance against WT-RBD (a,,c,e) and BA.5-RBD (b,d,f) are shown for undepleted (grey, light circles), mock-depleted (grey, full cycles), WT-RBD-depleted (red), and BA.5-RBD-depleted (blue) serum pools from the pre-WT/BA.5 (a,b), post-WT/BA.5 (c,d) groups, and BT-BA.5 (e,f).

 **Supplementary Figure 2: Percentage removal of WT- and BA.5-RBD–specific antibodies following homologous RBD depletion.**
(a) Percentage removal of WT-RBD reactivity after depletion with WT-RBD.
(b) Percentage removal of BA.5-RBD reactivity after depletion with BA.5-RBD.
Each pool consists of equal-volume contributions from the individual donors listed in Table 1. Each data point represents the percent reduction in ELISA IgG binding compared to mock-depleted control pools. The dotted horizontal line indicates near-complete depletion (≥ 98%). Vertical dashed lines separate vaccination-only groups from breakthrough infection groups. WT, wild type; BT, breakthrough infection.

**Supplementary Table 5:** Percentage removal of WT- and BA.5-RBD–specific antibodies following homologous and heterologous RBD depletion.

| **pool** | **% WT-RBD depleted** | | **% BA.5-RBD depleted** | |
| --- | --- | --- | --- | --- |
|  | **WT-RBD depleted pool** | **BA.5-RBD depleted pool** | **WT-RBD depleted pool** | **BA.5-RBD depleted pool** |
|  | ELISA AG: WT RBD | ELISA AG: BA.5 RBD | ELISA AG: WT RBD | ELISA AG: BA.5 RBD |
| pre-WT | > 98% | 78.2 | > 98% | > 98% |
| post-WT | > 98% | 86.1 | > 98% | > 98% |
| pre-WT/BA.5 | > 98% | 65.7 | > 98% | > 98% |
| post-WT/BA.5 | > 98% | 75.0 | > 98% | > 98% |
| BT-BA.1 | > 98% | 87.8 | > 98% | > 98% |
| BT-BA.2 | > 98% | 85.7 | > 98% | > 98% |
| BT-BA.5 | > 98% | 95.0 | > 98% | > 98% |
| prim-WT | > 98% | 64.1 | > 98% | > 98% |

**Supplementary Figure 3:** **Impact of RBD-specific antibody depletion on BA.5 neutralization capacity.**
Serum pools were depleted with WT-RBD (red) or BA.5-RBD (blue) and subsequently tested for residual neutralizing activity (NT50) against BA.5 spike–pseudotyped particles. Each pool represents equal-volume contributions from the individual donors listed in Table 1. Error bars represent the standard error of the mean (SEM) of at least two independent experiments. Dashed horizontal line indicates the assay cut-off. No statistical comparisons were performed.
